# Supplementary material for: Self-reported health, neuropsychological tests and biomarkers in fully recovered COVID-19 patients vs patients with post-COVID cognitive symptoms: A pilot study
Source: PLoS One. 2025 May 15;20(5):e0315486. doi: 10.1371/journal.pone.0315486 (PMC12080821; doi:10.1371/journal.pone.0315486)
Supplement: Attachment — Submitted filename: Response to Reviewers.docx [file pone.0315486.s003.docx]

**Response to Reviewers**

**PONE-D-24-53401**: Self-reported health, neuropsychological tests and biomarkers in fully recovered COVID-19 patients vs patients with post-COVID cognitive symptoms: a pilot study

Reviewer #1: The authors present a very insightful pilot study on the difference in mood, quality of life, and neuro-cognitive scores, and biomarkers between COVID-19 survivors with persistent cognitive symptoms and fully-recovered COVID-19 patients using the case-control design. The pilot study contributes additional exploration on the post-COVID-19 phenomenon to the existing published studies by recruiting laboratory-confirmed COVID-19 survivors as controls and by including both cognitive batteries and biomarkers as outcome measures. I do have some minor recommendations for the authors:

1. The difference between the ‘fully recovered COVID-19 patients’ and ‘patients with post-COVID cognitive symptoms’ groups is very arbitrary and highly reliant on single self-report. The authors are enjoined to specify whether they use the United Kingdom National Institute for Health and Care Excellence clinical case definition of post-COVID-19 condition or the World Health Organization 2021 Delphi Study case definition (accessible via <https://iris.who.int/bitstream/handle/10665/345824/WHO-2019-nCoV-Post-COVID-19-condition-Clinical-case-definition-2021.1-eng.pdf?sequence=1> ).
Response: Our thanks to the reviewer for this important comment. In the Introduction, we cite Scott et al (2024), stating that a widely accepted case definition and associated symptom timeframe is still under development. We therefore did not base our definition of long COVID on any one of the definitions developed by organizations such as NICE or WHO. We now review those definitions briefly and explain that we developed our own criteria (as most other studies to date have done, see Yang et al 2024) due to our specific focus on brain fog and other neurocognitive symptoms. The new text is included at the end of the paragraph under Study Participants. Please note that additional references have been added to the reference list.

2. It would be ideal to relocate the statement on line 130 “The sample was comprised of a total of 17…” to the Results section. The authors are also encouraged to at least declare if there was an attempt to reach a specific sample size for the pilot study involving continuous variables- the flat rule or the stepped rule of sample size determination for pilot studies.

Response: The sentence, “The sample was comprised of a total of 17 participants who had previously contracted COVID-19,” has been moved to the first line of the Results section.

We also appreciate the question regarding sample size estimation. We used the flat rule of thumb in sample size determination, with an aim of 30 participants, 15 in each group. This information has now been added in a new paragraph, Sample Size, in the Study participants section of the Methods. We have also added discussion regarding the difficulty in recruiting participants in the Limitations section.

3. It would be prudent to state why the other biomarkers mentioned in the review - GFAP, IL 12, IL 17, CRP, and TNF alpha - were not included in the pilot study.

Response: Our study protocol was finalized and approved by the Institutional Review Board in late 2021. The studies reporting on IL2 (Lai et al 2023), IL12, IL17 (Queiroz et al 2022) and GFAP (Vrettou et al 2024) were all published while our study was being conducted and therefore too late to influence our protocol. We did, however measure TNF alpha, as well as pTau 181 and pTau 217. Levels of TNF-alpha and pTau 181 were too low to be detected in our sample and were therefore not reported. Levels of pTau 217 were only detected in 2 individuals, one from each group, with levels higher in the Long-COVID individual. This information was inadvertently omitted from the original version of our manuscript. We now report this information in the Biomarker Analysis section of the Methods and in the Results section on blood biomarkers.

CRP was not included in our biomarker battery because we focused on inflammatory markers (IL-6, IL-10, TNF-alpha) that have been associated with poor cognitive function (Alvarez et al., 2007; Tegeler et al 2016; Wennberg et al 2019). This is now explained in the biomarker analysis section of the Methods. Please note that these references have been added to the reference list.

4. The authors are enjoined to use the Vancouver citation style wherein the in-text citation entails a superscript number after the period in compliance with the PLOS ONE submission guidelines.

Response: Our reference list follows the Vancouver style. However, citations in the text are in brackets, in keeping with the citation style of current articles in PLOS ONE. Please confirm if this is correct or needs to be revised to superscripts in the text.

Reviewer #2: The study fills important knowledge gaps in the elucidation of COVID-19 as a disease. The novelty this study brings is the rigorous comparison between prior lab-confirmed COVID-19 patients that have recovered (i.e., asymptomatic) versus lab-confirmed COVID-19 patients that have developed cognitive symptoms at least 6 months since diagnosis. The comparison spans cognitive and psychological function, perceptions of functional status and quality of life, and serum biomarkers of stress, inflammation, and neuroplasticity. The main weakness is the limited sample size.


Major
- In lines 74–76, the authors talk about post-COVID cognitive symptoms having higher frequency among individuals who had mild infection and who have a history of cognitive difficulties and depressive disorder. However, in the methodology, the authors do not include information on whether the cohort they recruited did have these factors. Knowing this clinical information is important as these conditions could confound the analysis. For example, a person who might have history of cognitive difficulties may fare worse in the neurocognitive function tests. How about vaccination history?

Response: We have expanded the text under Study participants and recruitment, explaining that all study participants were screened for a range of symptoms, including attention deficits, breathing difficulties, changes in mood, difficulties problem solving, fatigue, loss of smell, loss of memory, mental fogginess, muscle aches, paresthesia/pins and needles, slow processing speed, or other symptoms. While we did assess for changes in mood, we did not assess for preexisting mood disturbances, as these are common in the general population (<https://www.nimh.nih.gov/health/statistics/major-depression>; https://www.nimh.nih.gov/health/statistics/any-anxiety-disorder) and associated with higher rates of long COVID (Kataoka et al 2023).

The Study participants and recruitment section now includes a paragraph on exclusion criteria, where we now state that vaccination status was neither an inclusion nor an exclusion criterion. However, vaccination status and date of vaccination were recorded for all participants.

- For study participants, did the authors set any exclusion criteria? Since the analysis assesses cognitive and psychological functioning, quality of life, and serum biomarkers, confounders such as history of neurological and psychological conditions, any disabilities, or any immunological conditions might impact the data.

Response: A paragraph describing the exclusion criteria has been added to the study participants and recruitment section of the Methods. Participants with preexisting neurologic illness (i.e., stroke, tumor, head injury, or preexisting cognitive complaints) were excluded, which mitigated potential confounding factors for measurements of neuropsychological test performance and inflammatory/degenerative blood biomarker levels. In addition, adults who were unable to consent, pregnant women, prisoners, and anyone who reported having received monoclonal antibody treatment or COVID convalescent plasma for COVID-19 were also excluded.

Minor
- In the introduction (line 56), the authors define Post-COVID conditions (PCC) as a syndrome characterized by symptoms that extend well beyond the initial recovery period. The statement sounds arbitrary. Although the definition has not been standardized yet, perhaps the authors can include the usual time duration of recovery to make the statement less abstract and more clear.

Response: We have revised the sentence as follows: “Post COVID conditions (PCC) or long COVID, is a clinical syndrome characterized by symptoms of COVID-19 that extend well beyond the initial recovery period, a minimum of 12 weeks and often for a year or more.” Please also see our response to Reviewer 1’s first point above regarding new text in the Methods section regarding review of PCC definitions, most of which specify a 3-month minimum time period.

- How is “fully recovered patients” qualified? Since assessing recovery in this study relies on self report of symptoms, it is important to enumerate the review of systems asked (even just as supplemental information) so readers might be able to assess how comprehensive the assessment was.

Response: A description of the battery of current symptoms that all participants were asked to endorse (yes/no) is now provided in the Methods in the Study Participants and Recruitment section.

- Lines 111-113: What could be the reason why in ref 15, patients with PCC cognitive complaints did not differ from healthy controls on neurocognitive testing but tested WORSE than PCC without cognitive complaints?

Response: Thank you for this question and your careful reading of the text! The text on lines 111-113 was incorrect and has been changed to the following: “Patients with PCC cognitive complaints scored lower than healthy controls on neurocognitive testing but did not score worse than PCC patients without cognitive complaints.” By explanation, we further state that, “The authors concluded that all patients with PCC may experience cognitive difficulties, even though they may not express cognitive complaints.”

In addition, please note that the correct reference for that statement is not reference 15 Ariza et al. 2024 but is Ariza et al 2022 (new reference #17). This has been corrected in the reference list.

- The authors did not indicate the details on blinding and whether it was done in this study.

Response: Investigators were not blinded as to patient group in this study. This is now stated under study design.

- Lines 290-291: The authors should be careful with the use of the word “sustained” to describe the attenuation in neuroplastic activity especially since the data is not longitudinal.

Response: Thank you for this comment. The word “sustained” has been removed.

- Lines 291–292: What is the link between neuroplasticity and cognition? Is a decrease in neuroplasticity linked with cognitive problems? Which specific aspect of cognition has established links in literature? Perhaps the authors can include a concise statement on this.

Response: Decreased neuroplasticity has been associated with impaired cognition in both depressed patients as well as in older adults, especially regarding executive function. We have inserted a few lines stating this, lines 346-351. However, we further state that the relationship between the two is complex, and in terms of PCC likely affected by far-reaching effects from neuroinflammation (Lecca et al., 2022).

- Line 301: In the discussion by the authors, what may explain the discordance on IL-6 findings between the Lai et al. and Queiroz et al. studies?

Response: We have added text stating that the discordance is difficult to explain. Lai et al conducted a systematic review of 28 studies, while Queiroz et al studied a single cohort of 317 patients grouped into varying levels of COVID-19 infection. Queiroz did find higher levels of IL-6 among patients with a current, acute and severe form of COVID-19 illness. We have now added a substantial amount of text on explaining the discrepancy in the Discussion, lines 362-374.

- Lines 164–165: Consider reviewing the sentence for typographical errors.

Response: The error has been corrected to “-80 degree C freezer…”

- Line 191: Consider reviewing the case of the phrase “pro-inflammatory Index” (common vs. proper noun; consistency throughout the text)

Response: We now use Pro-inflammatory Index throughout the text.

- Lines 254-255: Consider reviewing the sentence, particularly on parallelism (“examine and compare self-reported and objective measures in PCC patients with cognitive complaints with…”)

Response: We have removed “examine and” from the sentence.

- Line 345-347: Consider putting the comma after the initialism AEO. Also consider reviewing the sentence. The authors are requested to clarify the sentence: Is it the AEO that is diminished in CFS patients?

Response: The comma has been removed and the sentence regarding AEO has been rewritten for clarity: Anhydrous Enol-Oxaloacetate (AEO) is a nutritional supplement which has anecdotally been reported to relieve physical and mental fatigue in Myalgic Encephalomyelitis/Chronic Fatigue Syndrome (ME/CFS) and long COVID patients.

- Line 349-351: Consider using an em dash instead of a hyphen.

Response: The hyphen has been replaced with an em dash.

- Line 354: The authors should be consistent in their use of the phrase “long COVID” and not hyphenate it. Also in line 358: “neuroinflammatory”

Response: Long COVID is no longer hyphenated throughout the manuscript. The hyphen has been removed in neuroinflammatory.
